# A Non-Contact and Real-Time Measurement Technique of Human Body Potential Using Electrostatic Induction Current Accompanied by Human Body Motion

**DOI:** 10.3390/s22197161

**Published:** 2022-09-21

**Authors:** Koichi Kurita

**Affiliations:** Department of Electrical Engineering and Computer Science, Faculty of Engineering, Kindai University, Higashiosaka 577-8502, Japan; kurita@hiro.kindai.ac.jp; Tel.: +81-82-439-1110

**Keywords:** human body potential, electrostatic induction, walking motion, non-contact detection, real-time detection

## Abstract

This paper describes a non-contact and real-time measurement technique of human body potential using ultra-sensitive electrostatic induction. When a participant moves his/her palm to a position approximately 30 cm away from an electrostatic induction sensor, electrostatic induction current flows transiently. It is clarified whether estimation of the human body potential is possible by simultaneously measuring the velocity of the participant’s palm and distance between the participant’s palm and sensor. In addition, even when the participant walks at a position approximately 50 cm away from the electrostatic induction sensor, it is confirmed that the estimation of human body potential is possible.

## 1. Introduction

It is well-known that the potential of the human body fluctuates with daily motion, such as during a walking motion. In general, digital electronic devices that make extensive use of high-speed, low-power integrated circuits are less resistant to electrostatic discharge (ESD). Therefore, an increase in human body potential often causes the destruction of digital electronic devices [1,2,3,4,5,6]. An increase in the potential of the human body is often caused by frictional charging, owing to daily motion, such as a walking motion. Many studies aimed at clarifying the mechanism of this phenomenon have been performed [7,8,9,10], and the mechanism of the human body potential increase has been clarified. If the potential of the human body rises to about 4 kV, due to the fluctuation of the potential of the human body, due to the movement of the human body, there is a risk that electrostatic discharge may occur. In these past studies, the measurement of human body potential was usually performed by bringing an electrode into contact with the human body [11,12,13]. Meanwhile, to continuously monitor the potential of the human body under non-contact conditions, an example has been proposed, in which a metal mesh induction electrode is installed on the ceiling of a room to measure the potential of the human body [14]. In addition, for managing electrostatic discharge in a factory, a surface voltmeter has been developed to detect the surface potential of a charged object under non-contact conditions. However, the detection distance of the surface voltmeter [15] is 3 cm, and its application is limited. In addition, an EPIC^TM^ sensor has been marketed as a potential measurement sensor in the past [16,17].

In this study, a method is proposed to measure human body potential under noncontact and real-time conditions, i.e., without contacting the human body with any device. Several studies on the detection of human movement using electrostatic induction have been made, and their usefulness has been shown [18,19,20,21,22]. Here, a new method of detecting a human body potential using electrostatic induction has been proposed. If this method can be realized, it will be possible to prevent malfunctions of precision electronic devices, owing to ESD, such as in an integrated circuit, as well as to prevent a fire or explosion, such as in a flammable atmosphere where volatile organic compounds (VOC) exist. To prevent these ESD failures, a demonstration of human potential measurement under non-contact conditions was conducted. Specifically, an electrostatic induction current transiently induced by human body movement was detected under non-contact conditions, at an electrode placed in the vicinity of the participant. At the same time, the distance between the sensor and human body, as well as the moving velocity of the human body, were measured by a distance measuring sensor module. Consequently, it was demonstrated that the human body potential could be detected under non-contact and real-time conditions. In this study, the human body potential measurement was performed under non-contact conditions from an electrostatic induction current obtained in each movement, and the participant’s moving palm in the vicinity of the sensor and walking movement were utilized as examples of human body movements.

In this article, Section 2 describes the detection principle of human body potential measurement using electrostatic induction. Section 3 describes experimental methods for measuring human body potential under non-contact conditions for palm movement and walking movement. Section 4 describes the experimental results and discussions obtained with the two methods. Section 5 describes a summary of this paper.

## 2. Detection Principle

### 2.1. Overview of the Inductive Current Detection Sensor

**Figure 1:**
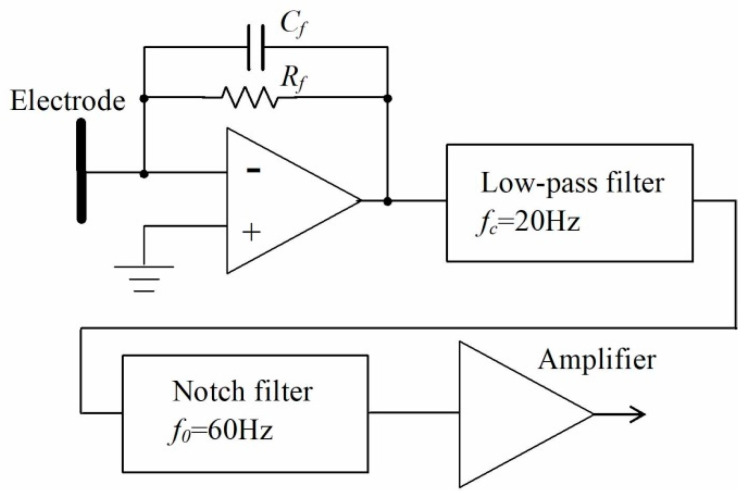
Block diagram of an ultrasensitive electrostatic induction sensor.

The induced current of the electrode placed in the sensor was measured with an *I*-*V* converter, with a feedback resistance of 500 MΩ of an operational amplifier, in which its non-inverting terminal is connected to the ground. Therefore, the electrode potential V = 0 was set. The offset voltage of the OP amplifier (LMP7721 by Texas Instruments, Dallas, TX, USA) used in this research is ±26 μV, and the input offset current is 6 fA. The details of the detection device are described in reference [23], so the details will not be described again in this paper. The current electrostatically-induced human body motion is usually very weak and, therefore, affected by electromagnetic noise from commercial power. Therefore, noise caused by commercial power was removed using a low-pass filter with a cutoff frequency of 20 Hz. The size of the electrode that induces the electrostatic induction current used in this study is 0.5 cm square. Because the electrostatic induction sensor manufactured in this research is a differential sensor, it does not affect the measurement, unless the charge on the objects around the sensor changes rapidly. The electrostatic induction sensor is shielded with a grounded metal, except for the 10 mm diameter aperture for the electrode. Figure 1 shows a block diagram of an ultrasensitive electrostatic induction sensor.

When the electrostatic induction sensor is placed near the human body, a capacitance is formed between an electrode inside the electrostatic induction sensor and the human body. In such a case, the electrostatic induction current is detected in the following two cases. One of them is that the electrostatic induction current is detected when the capacitance changes, due to the movement of the human body. The other is that the electrostatically induced current is detected when the potential of the human body fluctuates, for example, due to walking motion. The purpose of this research is to estimate the potential of the human body by detecting the current electrostatically induced by the change of the capacitance, due to the movement of the human body. Therefore, the speed of human motion was measured using two distance measurement sensors simultaneously, with the measurement of current electrostatically induced by human motion. Details of the detection method are provided in Section 2.2 and Section 2.3.

### 2.2. Human Body Potential Measurement Method by Palm Movement

**Figure 2:**
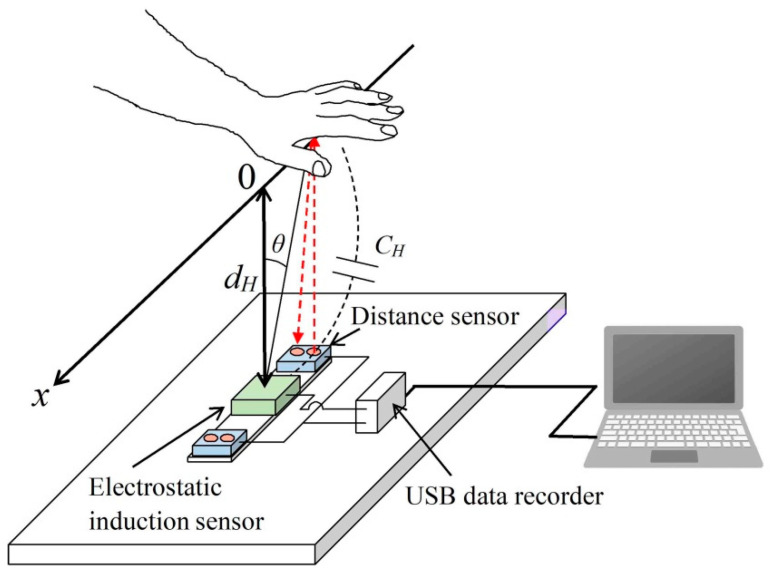
Schematic representation of the current electrostatically induced by palm movement.

As shown in Figure 2, when the palm of a person is at the origin of the *x*-axis, a case is considered where there is an electrode for measuring the electrostatic induction current inside the sensor, at a position vertically separated from the palm by a distance *d_H_*. It is assumed that the palm moves on the *x*-axis, with a direction parallel to the electrode plane as the *x*-axis. The origin on the *x*-axis is defined as a position where a line extending perpendicularly to the *x*-axis from the electrode of the electrostatic induction sensor is orthogonal. When the palm is at a position other than the origin on the *x*-axis, the angle between the perpendicular drawn from the origin on the *x*-axis to the electrode, and the straight line connecting the palm and electrode is *θ*. In addition, *ε*_0_ is the dielectric constant. Assuming that the electrode area of the electrostatic induction current sensor is *S_E_*, the capacitance *C_H_* formed between the palm of person and electrode can be expressed by the following Equation (1). Here, the electrode size on the side of the electrostatic induction sensor is 0.5 cm square, and the distance between the electrode and palm is usually approximately 30 cm. Therefore, in consideration of the fact that the distance between the electrode and palm is sufficiently large, as compared to the size of the electrode, the capacitance is approximated to be proportional to the electrode area *S_E_*.

(1) $$C_{H}\propto \frac{S_{E}}{{\left(d^{2}+x^{2}\right)}^{1/2}}\varepsilon_{0},$$

Then, the charge *Q* induced to the electrode is given by the following equation.

(2) $$Q=C_{H}(U_{B}-V),$$

Here, *V* is 0 V because it is a virtual ground. If the subject performs, for example, a stepping action while the palm is in motion, *d*(*U_B_ − V*)/*dt* = 0 is not satisfied because the human body potential *U_B_* changes. However, *d*(*U_B_ − V*)/*dt* = 0 holds if the subject moves only the palm, without moving his or her foot. Therefore, the electrostatic induction current *I_H_* flowing transiently to the electrode by electrostatic induction is given by the following equation. When capacitance is formed between the charged human body and electrode, a charge is induced in the electrode, but the potential *V* of the electrode becomes a virtual ground, due to the negative feedback of the operational amplifier. Regardless of whether or not there is a human body near the electrodes, the voltage of the electrodes can be regarded as a virtual ground if the operational amplifier is operating normally.

Because the potential *V* of the electrode is zero, the above equation can represent the charge *Q* induced on the electrode by the product of the capacitance *C_H_* formed between the electrode and palm and human body potential *U_B_*. Thus, for example, if the human body is grounded, then *Q* will be zero.

(3) $$I_{H}=\frac{dQ}{dt}=(U_{B}-V)\frac{dC_{H}}{dt}\propto (V-U_{B})\varepsilon_{0}\frac{S_{E}\cdot d_{H}\cdot x}{{\left(d_{H}^{2}+x^{2}\right)}^{3/2}}\cdot \frac{dx}{dt},$$

This equation indicates that the electrostatic induction current is proportional to the coordinates of the palm position on the *x*-axis and the palm velocity (*dx*/*dt*) = *v_H_*. In general, because the potential *V* of the electrode is smaller than the potential *U_B_*, (*V − U_B_*) takes a positive value when the potential of the human body is negatively charged.

Here, because the electric potential *V* of the electrode of Equation (3) is 0 V, the potential *V* can be ignored. Therefore, when the palm is initially at negative coordinates, the value of the peak *I_HP_* of the electrostatic induction current detected before and after the palm passes the origin is given by the following equation.

(4) $$I_{HP}\propto \frac{U_{B}S_{E}}{d_{H}^{2}}\cdot \frac{dx}{dt},$$

The approximation Equation (4) is based on the idea that the positive and negative peaks of the static induction current induced by the movement of the palm occur near the shortest distance *d_H_* from the sensor. Equation (4) is a rough approximation and not obtained by transforming Equation (3).

Accordingly, from the above equation, the human body potential *U_B_* is given by the following equation.

(5) $$U_{B}\propto \frac{I_{HP}}{\left(\frac{dx}{dt}\right)}d_{H}^{2}=\frac{I_{HP}}{v_{H}}d_{H}^{2},$$

**Figure 3:**
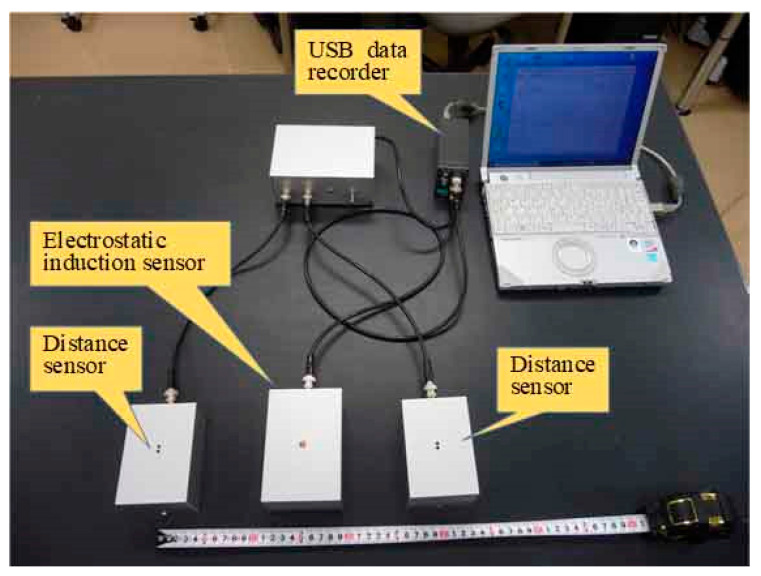
Photograph of the sensor system for the measurement of the electric potential of the human body by palm movement.

This equation indicates that the human body potential *U_B_* is proportional to the electrostatic induction current and inversely proportional to the product of the palm velocity *v_H_* and distance *d_H_* from the electrode to the palm. Therefore, it is shown that the potential of the human body can be estimated from the current electrostatically induced by the palm movement, palm velocity *v_H_*, and distance *d_H_* between the palm and electrode. Figure 3 shows a photograph of a system that measures human body potentials in real-time and non-contact, based on palm movements.

### 2.3. Human Body Potential Measurement Method by Walking Motion

**Figure 4:**
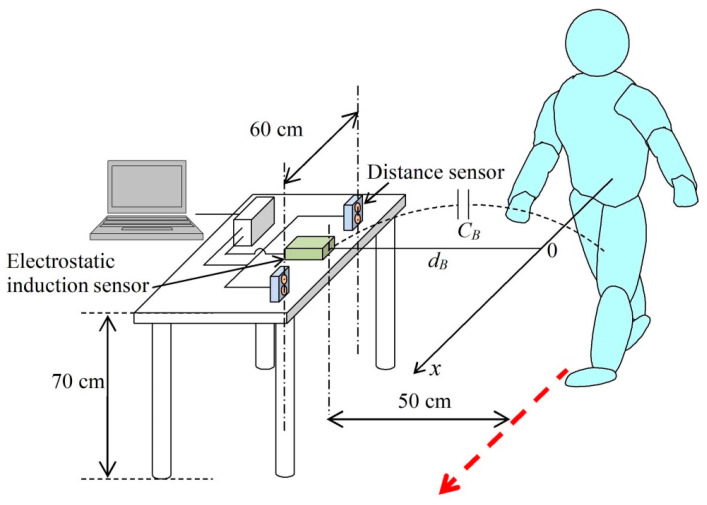
Schematic representation of the current electrostatically induced by walking motion.

As in the case of the movement of the palm, an electrostatic induction current is also detected when the participant passes by the vicinity of the electrode in a walking motion. As shown in Figure 4, the sensor for detecting an electrostatic induction current (with area *S_E_*) is placed in the vicinity of the human body, and a capacitance is formed between the electrode and human body [23,24,25,26,27]. Under the same assumption as in the case of measuring the human body potential by the movement of the palm, the electrostatic induction current *I*, transiently flowing to the electrode by the walking motion, is given by the following equation.

(6) $$I=\frac{dQ}{dt}=\frac{dU_{B}}{dt}C_{B}+(U_{B}-V)\frac{dC_{B}}{dt}\propto \frac{dU_{B}}{dt}C_{B}+(V-U_{B})\varepsilon_{0}\frac{S_{E}\cdot d_{B}\cdot x}{{\left(d_{B}^{2}+x^{2}\right)}^{3/2}}\cdot \frac{dx}{dt},$$

Here, *d_B_* is the length of a perpendicular extending from the origin of the *x*-axis to an electrode for measuring electrostatic induction current inside the sensor, and *C_B_* is the capacitance between the human body and electrode for the detection of induced current. Here, *V* is 0 V because it is a virtual ground; therefore, *d*(*U_B_ − V*)/*dt* can be replaced with *dU_B_*/*dt* in Equation (6).

The right side of the Equation (3) and second term of the right side of the Equation (6) are essentially the same. However, in the case of the walking motion, a term representing the electrostatic induction current resulting from the human body potential fluctuation, due to the walking motion, exists in the first term of the right side of the Equation (6). In both palm and walking motions, when the subject approaches the sensor electrode, the time integration of the trace of the induced current to the origin of the *x*-axis is the maximum value of the *Q* of the charge induced on the electrode.

It is well-known that the potential of the human body in a walking motion fluctuates in the walking cycle at the timings of contact and separation of the left and right feet. The first term of the right side of Equation (6) is an electrostatic induction current generated by the fluctuation of the human body potential *U_B_*, owing to the walking motion. The second term of the right side of Equation (6) is a current electrostatically induced when moving the human body at the moving velocity (*dx*/*dt*) = *v_B_*. Here, because the first term of Equation (6) has a frequency component that fluctuates in the walking cycle, it has a frequency component higher than that of the second term, i.e., the term reflecting the moving velocity. Therefore, by removing the electrostatic induction current of the first term on the right side of Equation (6) using a low-pass filter, it becomes possible to detect only the electrostatic induction current caused by the moving velocity of the human body.

Therefore, as in the case of the movement of the palm, the peak *I_HP_* of the electrostatic induction current detected by the movement of the palm in Equation (5) can be replaced with the peak *I_BP_* of the electrostatic induction current detected from the walking movement. Therefore, the potential of the human body can be estimated from the electrostatic induction current and walking speed of the second term on the right side of Equation (6), as well as the distance between the electrode and the human body, by using the following equation.

As will be shown in a later section, when the human body moves at a speed of about 0.3 m/s, the detection time width of the electrostatic induction sensor obtained in the experiment is about 4.8 s. The length of the walking path detected by the electrostatic induction sensor is estimated to be 1.44 m. Therefore, the frequency component of the electrostatic induction current, due to the movement of the human body, is about 0.43 Hz. On the other hand, since the time per step of walking is about 0.3 s, the frequency of walking motion is 3.3 Hz. Therefore, the electrostatically induced current detected by walking motion and electrostatically induced current caused by the movement of the human body can be separated by an LPF with a cutoff frequency of 0.98 Hz.

(7) $$U_{B}\propto \frac{I_{BP}}{\left(\frac{dx}{dt}\right)}d_{B}^{2}=\frac{I_{BP}}{v_{B}}d_{B}^{2},$$

**Figure 5:**
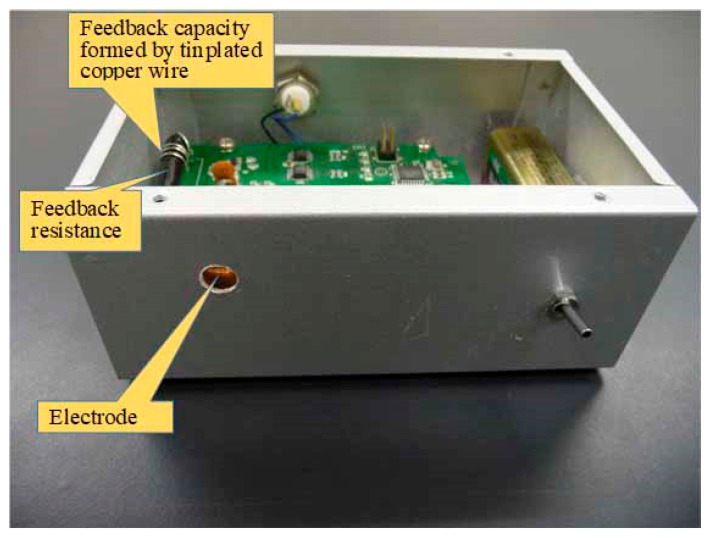
Photograph of the sensor for the current electrostatically induced by walking motion.

**Figure 6:**
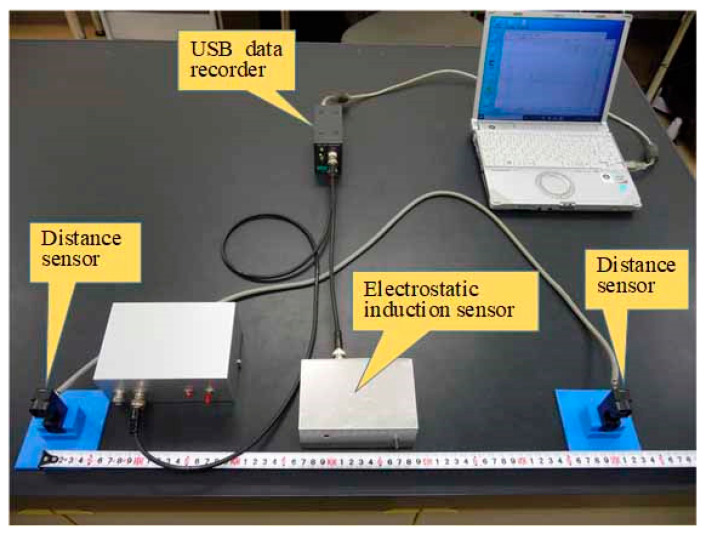
Photograph of the sensor system for the measurement of the electric potential of the human body by walking motion.

Figure 5 shows a photograph of the electrostatic induction sensor with the top cover open. Figure 6 shows a photograph of a system that measures the human body potential in real-time and non-contact by walking.

During walking, the human body potential changes with the walking cycle because the capacitance measured between the floor material and foot changes. The electric charge on the human body is gently charged by the triboelectrification caused by walking. The charge amount does not change with the walking cycle. It is known that the charge of a human body standing on a high-resistance floor material changes slowly. For example, the experimental value shown in Ref. [10] has a half-life of about 244 s. In this study, the measurement time is about 6 s, so the decrease in the human body charge can be ignored.

As the charged human body approaches the electrode, the capacitance between the human body and electrode increases. In contrast, the capacitance decreases as the human body moves away from the electrodes. This behavior causes positive and negative peaks in the electrostatically induced current and has important implications. Theoretically, the absolute values of these peaks should be the same, but the experimental values are slightly different, so the average of the two values is calculated. Another algorithm, a method using the trace area of the electrostatic induction current, is also conceivable, but it was not used in this study.

## 3. Experimental Method

### 3.1. Human Body Potential Measurement Method by the Movement of the Palm

The current electrostatically induced by the palm movement was detected under non-contact conditions using the sensor described in the previous section. At the same time, two infrared range sensor modules (GP2Y0E03 manufactured by Sharp, Sakai, Osaka, JAPAN) were used to measure the movement velocity of the palm and distance from the sensor. As shown in Figure 2, these sensor modules were installed 15 cm away from the center of the electrostatic induction current sensor. The distance measurement range of these sensor modules was 4 to 50 cm, and 0 V was set to be output when the palm of the participant was not within the distance measurement range. The distance measuring sensor was shielded by a grounded metal, except for the 10 mm × 6 mm rectangular aperture, which was the infrared LED emitting/receiving unit. The electrostatic induction current sensor and distance measurement sensor modules were measured at a sampling frequency of 200 Hz by a USB data recorder (DACS 15BXP manufactured by Dacs Giken, Akaiwa, Okayama, JAPAN). Thus, the two ranging sensor modules were spaced 30 cm apart. When the palm of the participant moved on the *x*-axis, the time required to move the 30 cm could be detected from the time difference Δ*t* between the rise times of the signals of the two distance measurement sensor modules. Therefore, the velocity of movement of the palm could be determined from the time difference Δ*t* and distance between the distance measurement sensors. Moreover, the distance to the palm could be measured simultaneously by the two distance measurement sensor modules. The wiring of the distance measurement sensor modules needs measures to protect against electrostatic induction noise.

Therefore, by performing the measurements shown in Figure 2, it is possible to simultaneously obtain the electrostatic induction current *I_HP_* induced by the motion of the palm, velocity (*dx*/*dt*) of the palm, and distance *d_H_* between the palm and electrode. By substituting the data obtained by the measurement into Equation (4), it becomes possible to estimate the potential of the human body of the participant, with the simple act of moving the palm of the participant.

### 3.2. Human Body Potential Measurement Method by Walking Motion

The current electrostatic induced by the walking motion was detected by the measurement arrangement shown in Figure 4. Two infrared range sensor modules were used to measure the walking speed and distance between the sensor and human body, as in the case of palm movement. As shown in Figure 4, when the participant walked on the *x*-axis, the time required to move 60 cm could be detected from the time difference Δ*t* between the rising of the signals of the two distance measurement sensor modules. However, the detected electrostatic induction current includes the signal from the walking motion of the first term on the right side of Equation (5). This signal is generated when the human body potential fluctuates in the walking cycle and has a frequency component higher than the current component caused by the walking speed. Therefore, by measuring the electrostatic induction current signal with LabVIEW and filtering it using a low-pass filter, only the electrostatic induction current *I_B_* owing to the moving velocity of the human body was detected. The cutoff frequency of the low-pass filter used in this research was 0.98 Hz. By substituting the data obtained by the measurement into Equation (7), it is possible to estimate the human body potential of the participant by merely measuring the walking motion of the participant.

### 3.3. Experimental Procedure

Friction between natural rubber and pulp produces a negative triboelectric charge on the surface of the natural rubber. In this study, the human body potential was changed from 0 V to approximately negative 5 kV by rubbing the negative charge of this natural rubber on the human body. The human body potential was measured using a contact-type human body potential measuring instrument (MODEL KSD-4100 manufactured by Kasuga Denki, Kawasaki, Kanagawa, JAPAN), immediately before and after the measurement. The average value of the human body potential before and after the measurement was regarded as the human body potential during the experiment.

The human body potential measurement proposed in this study is not intended to measure the fluctuation of the potential that changes with the movement of the human body while walking. The purpose of this study is to detect the steady-state human potential of a subject under non-contact conditions. In human body potential measurement by the movement of the palm, because the time for moving the hand is as short as 2 s or less, the human potential measured using the KSD 4100 was almost the same before and after the palm movement. However, in the case of the walking motion, the walking motion for about 10 s was measured. Unfortunately, the human body and clothes may come into contact during the walk, and the human body may be charged; therefore, human potential measured using the KSD 4100 may differ greatly before and after the walking movement. Therefore, only data with a difference of 5% or less in human potential before and after walking was used for analysis.

The experiments were carried out in a laboratory with a floor of PVC material. The participant was a healthy male (22 years old), and the participant’s footwear was a sports shoe made of rubber. The subject was 169 cm tall, weighed 57 kg, and was of a lean build. The experiments were conducted in a laboratory at a temperature range of 23.6 to 26.8 °C (average temperature 25.9 °C), with a relative humidity of 48% to 52% (average relative humidity 49%).

In the measurement of human body potential by the movement of the palm, as shown in Figure 2, the participant’s left palm was moved from left to right, in order to simultaneously detect the electrostatic induction current trace and distance measurement signal. At that time, the participant was in a standing position. In human body potential measurement by the walking motion, the participant’s natural-paced walking motion was detected from a position 3 m away from the sensor. Using the measurement arrangement shown in Figure 4, the electrostatic induction current trace and distance measurement signal at the time of the walking motion were simultaneously detected.

## 4. Experimental Results and Discussion

### Relationship between Current Electrostatically Induced by Palm Movement and Human Body Potential

**Figure 7:**
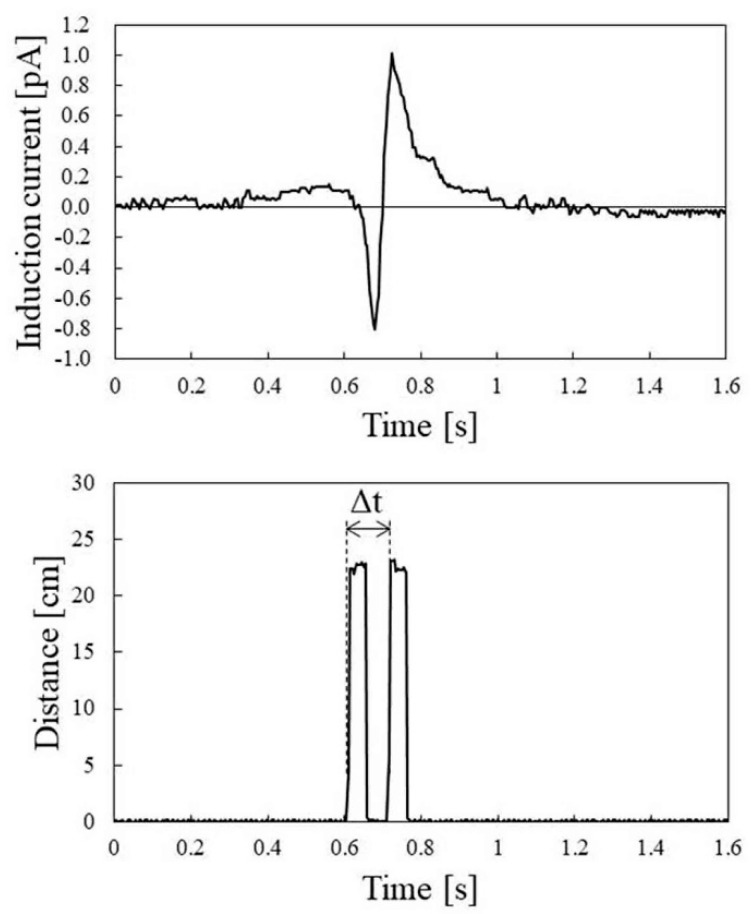
Electrostatic induction current trace induced by palm movement (**upper panel**) and ranging trace obtained by ranging sensor module (**lower panel**).

Figure 7 shows a typical electrostatic induction current trace induced by palm movement. This trace is the result of the detection at a human potential of 0.6 kV. As expected from Equation (3), the electrostatic induction current trace had a negative peak; then, passed through 0 and a positive peak. When the human body potential was negatively charged, the result could be qualitatively described as follows. Initially, the participant’s palm was at the minus coordinate of the *x*-axis and stationary. At this time, a capacitance was formed between the palm and electrode, and a positive charge was induced on the electrode. At that time, the electrostatic induction current did not flow to the electrodes, because the palm was stationary. However, if the distance between the palm and electrode was reduced, due to the movement of the palm, the charge induced on the electrode would increase, and a negative electrostatically induced current would flow to the electrode. When the palm passed the origin of the *x*-axis, the amount of increase in the positive charge on the electrode was zero, and the electrostatic induction current was zero. Then, as the distance between the palm and electrode increased, the positive charge induced by the electrode decreased, and the positive electrostatic induction current flowed.

As shown in Figure 2, the movement of the palm was measured by two distance measurement sensor modules, simultaneously with the measurement of the electrostatic induction current. Because the difference Δ*t* between the rise times of the signals from the two distance measurement sensor modules and sensor module spacing was 30 cm, the moving velocity of the palm could be determined. The traces in the lower panel of Figure 7 are the output traces of the two distance measuring sensor modules. From these traces, it can be seen that the moving velocity was 2.85 m/s, as the moving time of the palm was 0.105 s. In addition, it can be seen that the distance to the sensor was 0.224 m. Because the negative and positive peaks of the electrostatic induction current trace were −0.80 and +1.01 pA, respectively, *I_HP_*, which is the average value of the absolute values of these peak currents, was 0.91 pA. Accordingly, when the human body potential was 0.6 kV, the right side of Equation (5) was 1.425 pA·s/m^2^. The measurements of the electrostatic induction current and distance measurement data induced by the movement of the palm were performed multiple times, while changing the potential of the participant’s body.

**Figure 8:**
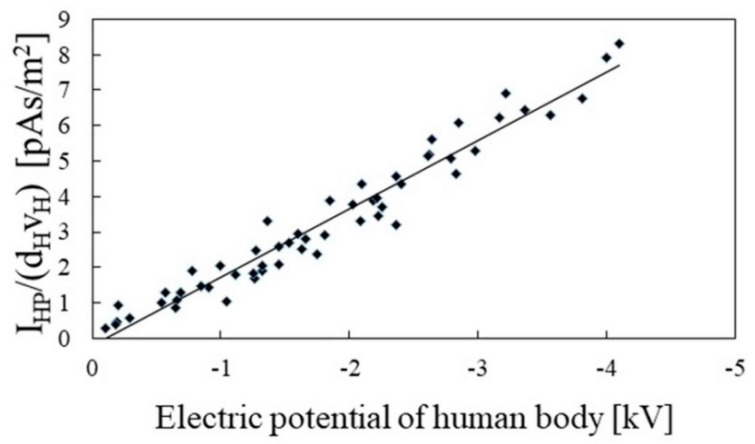
Human body potential dependence of the value of the peak value of the electrostatic induction current (*I_HP_*) trace induced by the movement of the palm divided by the product of the palm velocity (*v_H_*) and detection distance (*d_H_*).

Figure 8 shows the relationship between the right side of Equation (5) and human body potential. The horizontal axis of this figure is the potential of the human body, and the vertical axis is the peak value of the electrostatic induction current trace, divided by the product of the moving velocity of the palm and detection distance. The slope of the regression line, obtained based on these data, was 1.92. The Pearson’s correlation coefficient between the regression line and data was 0.97. These results indicate that the human body potential and right side of Equation (5) have a proportional relationship. Therefore, it was found that it was possible to estimate the human body potential from the electrostatic induction current trace detected by the movement of the palm.

**Figure 9:**
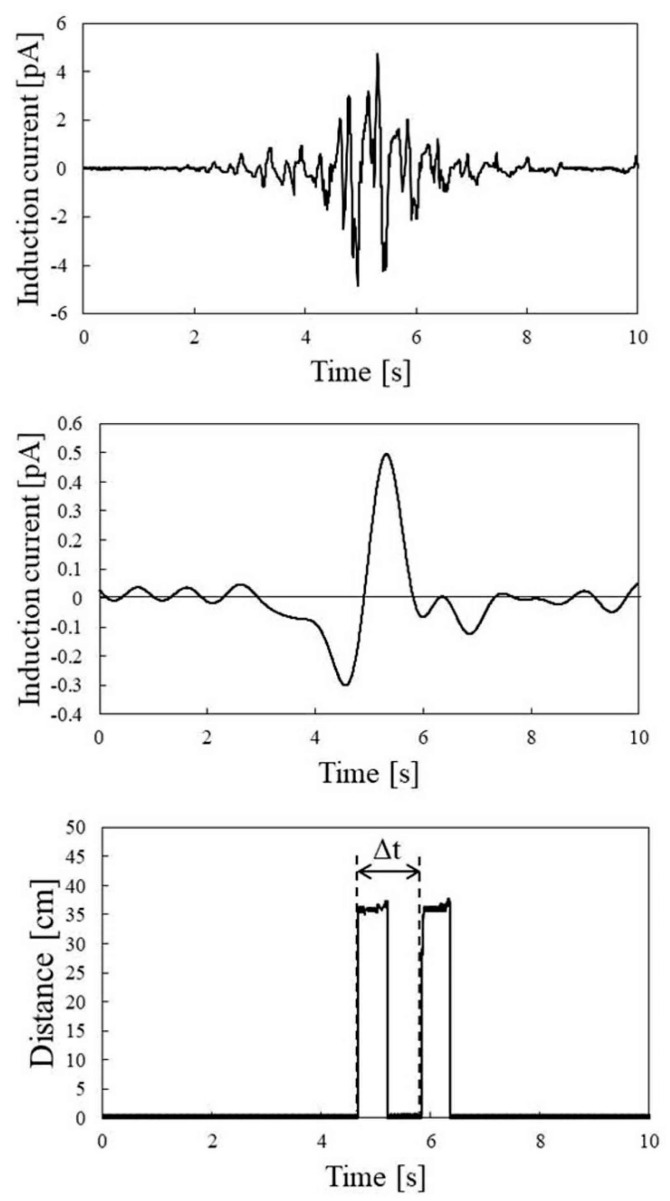
Electrostatic induction current trace induced by walking motion (**upper panel**), electrostatic induction current trace after passing low-pass filter (**middle panel**), and distance measurement trace obtained by distance measurement sensor module (**lower panel**).

Figure 8 shows a typical electrostatic induction current trace (upper panel) obtained from a walking motion, electrostatic induction current trace after passing through a low-pass filter (middle panel), and distance detection signal trace (lower panel) detected simultaneously. These traces are the result of detection at a human potential of 0.85 kV. In the upper panel of Figure 9, as predicted by Equation (6), a periodic trace, owing to the walking motion, is detected. In this electrostatic induction current trace, the appearance of the participant moving away after approaching the sensor by the walking motion is reflected in the intensity change of the amplitude of the walking signal. A trace obtained by removing the signal caused by the walking motion using a low-pass filter with a cutoff frequency of 0.98 Hz is shown in the middle panel of Figure 9. As shown in the second term on the right side of Equation (6), a negative peak was detected when the participant approached the electrostatic induction sensor, and a positive peak was detected when the participant moved away from the sensor.

**Figure 10:**
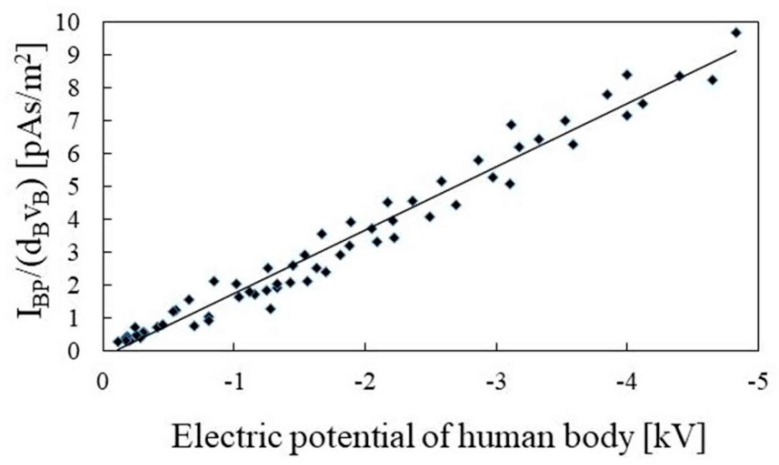
Human potential dependence of the peak value of the electrostatic induction current (*I_BP_*), trace induced by walking motion divided by the product of walking speed (*v_B_*), and detection distance (*d_B_*).

Meanwhile, from the trace of the distance measurement sensor module shown in the lower panel of Figure 9, it is possible to obtain the time for the participant to move the distance of 60 cm. From this trace, it can be seen that the walking speed of the participant was 0.52 m/s, because the time difference Δ*t* was 1.16 s. In addition, the distance to the sensor was 0.356 m. From the middle panel of Figure 9, the negative peak and positive peak of the electrostatic induction current trace were −0.30 pA and +0.49 pA, respectively. Therefore, *I_BP_*, which is an average value of the absolute value of the peak current, was 0.39 pA. Accordingly, when the human body potential was 0.85 kV, the right side of Equation (7) was 2.11 pA·s/m^2^. Figure 10 shows the result of examining the relationship between the right side of the expression in Equation (7) and human body potential, by changing the human body potential of the participant. The horizontal axis is the potential of the human body, and the vertical axis is the peak value of the electrostatic induction current trace divided by the product of the walking speed and detection distance. The slope of the regression line, based on these data, was 1.91. In addition, the Pearson’s correlation coefficient between the regression line and data was 0.93, and it has been experimentally shown that the human body potential was proportional to the right side of Equation (7). Therefore, the detection of the human body potential was possible from the electrostatic induction current trace detected from the walking motion.

When using a conventional electrostatic voltmeter, it is necessary to bring the sensor into contact with the subject, or to bring the sensor closer to 3 cm or less, when measuring the potential of the human body. By using the method proposed in this research, it is possible to estimate human potential under non-contact conditions, with a sensor several tens cm away from the subject. However, when measuring the human body potential by the motion of the palm, if the potential of the human body fluctuates greatly during the motion of the palm, accurate measurement of the human body potential becomes difficult. In the case of measuring the human body potential by the walking motion, if the friction between the hand and clothing is unfortunately generated during walking, accurate measurement of the potential of the human body becomes difficult.

For example, by placing the system proposed in this study at the entrance gate of a room that requires the prevention and control of electrostatic discharge, it becomes possible to detect the human body potential of the person who is trying to enter the room under non-contact conditions. It may be used as part of a system that automatically discharges electricity if the human potential of the person trying to enter the room exceeds a set reference value.

Although this paper presents data obtained from only one subject, preliminary experiments have obtained data from three subjects, and the results are similar to those in this paper. However, at this stage, there are insufficient data to discuss subject dependence. In the future, I plan to acquire data from more subjects and clarify subject dependence.

## 5. Conclusions

In this study, the electrostatic induction phenomenon accompanying human body movement was used to establish a non-contact and real-time measurement technique of human body potential. It was clarified that it is theoretically possible to estimate a human body potential by detecting the current electrostatically induced in an electrode placed near the human body, in accordance with the movement of the human body. In this research, whether it is possible to estimate human potential by using two examples of human motion, i.e., palm motion and walking motions, was verified. First, the simultaneous measurement of the current electrostatically induced by the movement of the palm of the participant, movement velocity of the palm, and distance between the palm and sensor were performed approximately 30 cm away from an electrostatic induction sensor. Consequently, it was determined that the potential of the human body can be estimated from the current electrostatically induced by the motion of the palm. Furthermore, the potential of the human body can be estimated by simultaneously measuring the current electrostatically induced when the participant walks at a position approximately 50 cm away from the electrostatic induction sensor, as well as the walking speed and distance between the human body and sensor.

## Data Availability

Not applicable.
